# Spatial variation in the climatic predictors of species compositional turnover and endemism

**DOI:** 10.1002/ece3.1156

**Published:** 2014-07-29

**Authors:** Giovanni Di Virgilio, Shawn W Laffan, Malte C Ebach, David G Chapple

**Affiliations:** 1 School of Biological Earth and Environmental Sciences, University of New South WalesSydney, New South Wales, 2052, Australia; 2 School of Biological Sciences, Monash UniversityClayton, Victoria, 3800, Australia; 3 Allan Wilson Centre for Molecular Ecology and Evolution, School of Biological Sciences, Victoria University of WellingtonWellington, 6140, New Zealand

**Keywords:** Biodiversity hotspots, climatic heterogeneity, conservation, geckos, macroecology, skinks

## Abstract

Previous research focusing on broad-scale or geographically invariant species-environment dependencies suggest that temperature-related variables explain more of the variation in reptile distributions than precipitation. However, species–environment relationships may exhibit considerable spatial variation contingent upon the geographic nuances that vary between locations. Broad-scale, geographically invariant analyses may mask this local variation and their findings may not generalize to different locations at local scales. We assess how reptile–climatic relationships change with varying spatial scale, location, and direction. Since the spatial distributions of diversity and endemism hotspots differ for other species groups, we also assess whether reptile species turnover and endemism hotspots are influenced differently by climatic predictors. Using New Zealand reptiles as an example, the variation in species turnover, endemism and turnover in climatic variables was measured using directional moving window analyses, rotated through 360°. Correlations between the species turnover, endemism and climatic turnover results generated by each rotation of the moving window were analysed using multivariate generalized linear models applied at national, regional, and local scales. At national-scale, temperature turnover consistently exhibited the greatest influence on species turnover and endemism, but model predictive capacity was low (typically *r*^2^ = 0.05, *P* < 0.001). At regional scales the relative influence of temperature and precipitation turnover varied between regions, although model predictive capacity was also generally low. Climatic turnover was considerably more predictive of species turnover and endemism at local scales (e.g., *r*^2^ = 0.65, *P* < 0.001). While temperature turnover had the greatest effect in one locale (the northern North Island), there was substantial variation in the relative influence of temperature and precipitation predictors in the remaining four locales. Species turnover and endemism hotspots often occurred in different locations. Climatic predictors had a smaller influence on endemism. Our results caution against assuming that variability in temperature will always be most predictive of reptile biodiversity across different spatial scales, locations and directions. The influence of climatic turnover on the species turnover and endemism of other taxa may exhibit similar patterns of spatial variation. Such intricate variation might be discerned more readily if studies at broad scales are complemented by geographically variant, local-scale analyses.

## Introduction

Reptiles are a group of conservation concern because they are especially sensitive to climatic variability (Gibbons et al. 2000; Wake 2007; Sinervo et al. 2010) and habitat change (Gardner et al. 2007; Ribeiro et al. 2009; Nuneza et al. 2010). Determining how they might respond to environmental variability could provide insights into the targeting and planning of their conservation. Climatic and habitat heterogeneity have been found to be predictive of reptile species richness (Schouten et al. 2009; Kutt et al. 2011) and species compositional turnover (Chen et al. 2011). In several cases, temperature-related variables have been shown to explain more of the variation in reptile distributions than precipitation or nonclimatic predictors (Rodriguez et al. 2005; Aragon et al. 2010; Qian 2010; Buckley et al. 2012).

However, these studies have focused on broad-scale or geographically invariant reptile-environment dependencies. This raises the question of how well the insights they generated extrapolate to different locations and spatial scales. Species–environment relationships can exhibit marked spatial variation (spatial nonstationarity) because the influence of environmental processes on biodiversity can vary with spatial scale and geographic location (Rahbek 2005; Buckley and Jetz 2008). For instance, effects such as climatic variability may be mediated at fine spatial scales by topographic variations over relatively small distances (O'Brien et al. 2000) with implications for how taxa might respond to such changes. Species–environment relationships can also vary between different locations, which is evident in reptile distributions in northeastern Australia (Powney et al. 2010) and the Iberian Peninsula (Moreno-Rueda and Pizarro 2009). Species-environment dependencies also exhibit anisotropic variation because ecological and physical patterns change with direction (Burley et al. 2012). This directional variation may be driven by strong environmental gradients (Hagen et al. 2008), habitat structure or species interactions (Haase 2001). Consequently, fundamental differences in the nature of reptile biodiversity–environmental turnover relationships may be obscured by broad-scale or spatially invariant analyses.

Species turnover is the rate of change in biological composition across geographic space and is a measure of species diversity (Wilson and Shmida 1984; Williams 1996; Vellend 2001). A high rate of species turnover indicates that the assemblages at two locations are different, whereas they are similar if the rate of turnover is lower. The rate of turnover will be low in areas where species composition is relatively uniform compared with higher turnover in the distributional breaks that delineate such areas (Walker et al. 2003). This is possibly because multiple species reach their geographic range limits at the location of a distributional break (Keith et al. 2013). A large, distinct low turnover zone may therefore indicate a geographic concentration of several species and possibly an area of high species diversity. Thus, measuring the continuous variation in species turnover across a landscape can quantify the gradual variation and disjunctions in species community composition (Williams 1996; Williams et al. 1999; Di Virgilio et al. 2012, 2013). Similarly, environmental turnover is the rate of change in physical conditions across an environmental gradient and is thus a proxy for environmental heterogeneity. For instance, topographic turnover will be low across a floodplain, but comparatively higher at an exposed rocky ridge. Relating turnover in environmental variables with species turnover and endemism can estimate the influence of environmental variability on these biotic patterns, and by implication, the variation in composition of species distributions across geographic space (Di Virgilio et al. 2012).

We assess the geographic and directional variation in the relationships between species turnover, endemism and turnover in different climatic variables using New Zealand reptiles as a case study. Specifically, how does the nature of these biophysical relationships change in different parts of New Zealand, and as a function of changing scale and direction? Moreover, species diversity and endemism hotspots may both be prioritized for conservation, but their spatial distributions differ for both mammals (Ceballos and Ehrlich 2006) and birds (Orme et al. 2005). We therefore also assess the geographic correspondences between reptile turnover and endemism hotspots and whether they are influenced differently by climatic turnover. We address these aims by quantifying the directional variation in reptile species turnover, endemism and turnover in climatic variables across New Zealand using moving window analyses. We then assess how the relationships between these biotic patterns and turnover in environmental variables vary with changing direction at a national scale and for different locations at regional and local scales. A secondary aim of this research is to determine whether associations between species turnover, endemism and climatic turnover differ according to biogeographic regions that are regarded as either species rich or poor.

## Materials and Methods

### Study area

**Figure 1:**
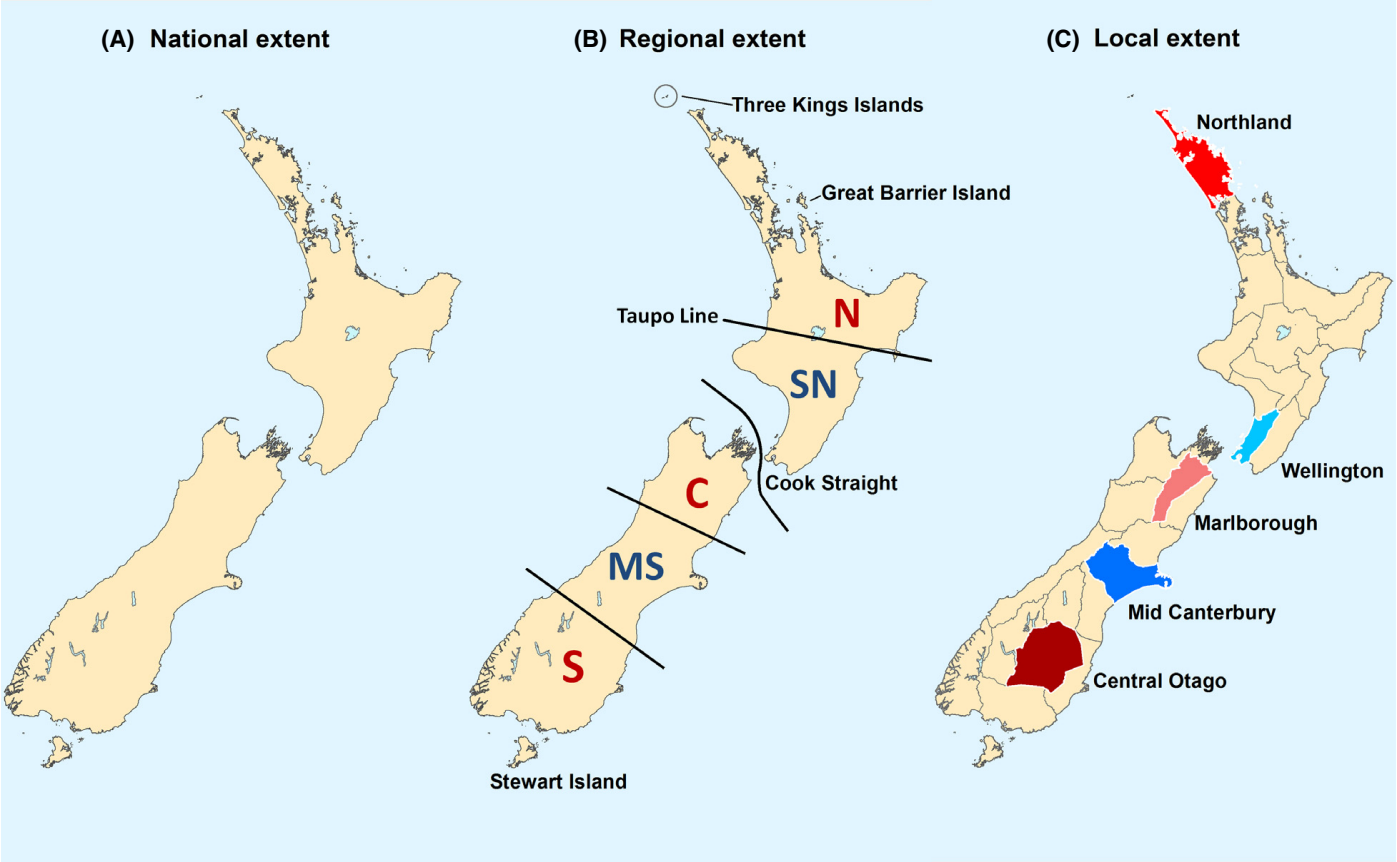
The varying spatial extents and different localities used to stratify the generalized linear model (GLM) analyses of reptile species turnover, corrected weighted endemism, and environmental turnover. National scale analysis across the whole country (A). Biogeographic regions used in the regional-scale analyses, with area codes: N, Northern North Island; SN, Southern North Island; C, Central New Zealand; MS, mid-South Island; S, Southern South Island (B) after (Gibbs 2006). Smaller extent areas used in local-scale analyses (C).

New Zealand is located in the southwestern Pacific Ocean and is approximately 267,530 km^2^ in area (Fig. 1). New Zealand's two main islands, the North Island (NI) and South Island (SI), are topographically complex, and span ∼12° of latitude and have a range of climatic gradients. The highest point in the Southern Alps is 3750 m above sea level, and this range divides the SI along its length, with a rugged coastline to the west and coastal plains to the east. The smaller NI is volcanic and comprises the Taupo volcanic plateau which delineates a biogeographic boundary at ∼39°S between the NI northern and southern regions. The climate of New Zealand varies from subtropical at its northern extent to mostly temperate in the SI. There is substantial climatic variation between regions, with high rainfall on the west coast of the SI, alpine conditions in mountainous areas, while the eastern plains are drier.

The New Zealand biota is highly endemic with marked variations in diversity and disjunctions in species ranges. Research on its reptiles has focused on their origins (e.g., Rest et al. 2003; Chapple et al. 2009; Jones et al. 2009; Nielsen et al. 2011), but comparatively less so on their distributional patterns. We examine reptile distributions across the North and South Islands plus islands within 100 km of their coastlines from 34.1°S to 47.3°S (a range of ∼1500 km).

New Zealand has been partitioned into five broad-scale biogeographic regions of varying diversity and endemism based on the inferred effects of historical climatic and geological processes (for a review see Wallis and Trewick 2009) and the distributions of plants (Wardle 1963, 1988; McGlone 1985; Rogers 1989; Connor 2002) and insects (Craw 1989; Gibbs 2006). The northern NI and northern and southern regions of the SI are characterized as having high diversity/endemism compared with lower diversity/endemism in the mid-SI and southern NI (Fig. 1B).

### Data sources

Reptile point locality data were derived from the Bioweb Herpetofauna Database (New Zealand Department of Conservation 2012) and updated by DGC to reflect the current taxonomy (Chapple et al. 2009; Hay et al. 2010; Nielsen et al. 2011; Hitchmough et al. 2013). Records with multiple observations of the same species at the same location and time were treated as single observations. Spurious records were removed and each species record was checked to confirm that it matched currently recognized species distributions. We excluded fossil/subfossil and translocated records. The revised database contained 10,477 reptile observations, comprising 53 species of skink (genus *Oligosoma*), 42 gecko species in seven genera (*Dactylocnemis, Hoplodactylus, Mokopirirakau, Naultinus, Toropuku, Tukutuku,* and *Woodworthia*), and the tuatara, *Sphenodon punctatus*. The species included in the study are not subspecies or regional variants; however, some are undescribed species that are yet to be classified, as the taxonomic status of New Zealand reptiles is rapidly evolving. Seventy-nine percent of records (*n* = 8315) had a positional accuracy between 1 km and 10 km.

We initially assessed the fourteen environmental data sets listed in Table 1 for potential inclusion as predictor variables by assessing their multicollinearity. These comprised elevation derived from the ∼85 m resolution Shuttle Radar Topography Mission (SRTM) Digital Elevation Model (DEM; Jarvis et al. 2008) and thirteen climatic surfaces at ∼1 km resolution derived from the Worldclim data sets (Hijmans et al. 2005). Nine of the climatic variables (BIO3 through BIO11) were strongly correlated with annual mean temperature (BIO1) and mean diurnal range (BIO2) and also with each other, so they were excluded. Only turnover in the remaining climatic variables had a marked effect on lizard turnover and corrected weighted endemism (CWE), so the elevation turnover variable was removed via backward elimination. Thus, the independent variables used in this study are turnover in annual mean temperature (BIO1), mean diurnal range (BIO2), annual precipitation (BIO12), and precipitation seasonality (BIO15).

**Table 1:** The four climatic data sets in bold font were used in this study. The remainder were excluded on the grounds of marked multicollinearity (BIO3–BIO11) or having a negligible influence on the response variables (elevation)

Data set	

Topography	Elevation
Climate	**Annual Mean Temperature (BIO1)**
	**Mean Diurnal Range (BIO2)**
	Isothermality (BIO3)
	Temperature Seasonality (BIO4)
	Max Temperature of Warmest Month (BIO5)
	Min Temperature of Coldest Month (BIO6)
	Temperature Annual Range (BIO7)
	Mean Temperature of Wettest Quarter (BIO8)
	Mean Temperature of Driest Quarter (BIO9)
	Mean Temperature of Warmest Quarter (BIO10)
	Mean Temperature of Coldest Quarter (BIO11)
	**Annual Precipitation (BIO12)**
	**Precipitation Seasonality (BIO15)**

The georeferenced species point occurrence and climatic data sets were each aggregated to separate sets of 4.5 km × 4.5 km cells. The analyse included cells in which no species had been recorded. Aggregating the data sets to coarser cell sizes up to 10 km produced similar patterns of results by the moving window analyses. For instance, the location of species turnover and endemism hotspots was consistent across resolutions, although they were rendered in less detail at 10 km. Generally, the rate of species turnover decreases as the cell size is increased.

### Spatial analyses

We measured the variation in reptile species turnover, endemism, sample redundancy, and climatic turnover across New Zealand using the moving window analysis method described by Di Virgilio et al. (2012). This method quantifies the directional variation in biotic distributions or physical data at any specified geographic extent and resolution. These analyses were implemented using the Biodiverse software, version 0.18 (Laffan et al. 2010).

A moving window comprised a mosaic of 4.5 km × 4.5 km cells and was divided into two neighborhood sets following Di Virgilio et al. (2012). One neighbor set (NS1) was defined as a straight line of cells, centered on the cell being processed. A larger neighbor set (NS2) was defined as a semi-ellipse and was orientated perpendicularly to NS1 and constrained to one side such that any derived metrics are unidirectional. The moving windows are then iteratively positioned over each cell in the study area in turn. At each iteration, the species or climatic values within the neighbor sets around a cell are used to calculate a species turnover, endemism, or a turnover score for each climatic variable for that cell. To capture the directional variation in species and climatic turnover and CWE, the moving window analyses were repeated with the window rotated through 24 different angles from 0° to 360°. The full set of moving window analyses were applied separately to each dataset.

The dimensions of the moving window determine the number of cells that comprise each neighbor set and consequently affect how spatial variation is quantified. To define the moving window dimensions, we derived a correlogram of the variation in reptile species turnover as a function of distance. A correlogram is a plot of the rate of change in species turnover values between pairs of sites with increasing geographic separation distance. The distance at which the turnover values start to plateau is the maximum distance to which there is spatially structured turnover. This range is the maximum distance for detecting changes in the rate of species turnover and its value was used to specify the length of the moving window. We used the same window size for all analyses, as we aim to compare the geographic variation in species turnover and endemism and the influence of climatic turnover on these patterns.

The Sørensen dissimilarity index (eqn 1) was used to quantify the compositional turnover of the reptile assemblages.


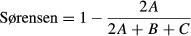
(1)

where *A* is the number of species found in both neighbor sets; *B* is the number of species unique to NS1; and *C* is the number of species unique to NS2. The Sørenson index ranges from 0 (neighbor sets are identical/no turnover) to 1 (neighbor sets share no values/complete turnover). Alternative compositional dissimilarity indices to the Sørensen metric were also trialled. The Jaccard index produced similar results (the underlying formula is similar to the Sørensen index). Bray Curtis is abundance weighted and so less appropriate for these data due to biases in sampling effort. The turnover patterns were less apparent in the results generated using Simpson's *β*. This suggests that part of the turnover quantified is turnover in species richness, but such richness gradients are important of themselves, and thus, the Sørensen metric was selected.

For the interval-scaled climatic data, a numeric dissimilarity index (eqn 2) was used to compare the set of numeric values in each neighbor set by calculating the mean absolute difference between all pairs of values in NS1 and NS2.


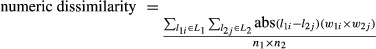
(2)

where *L*_1_ and *L*_2_ are the sets of climatic values in NS1 and NS2, respectively, *w*_1*i*_ and *w*_2*j*_ are the abundances of climatic values *l*_1*i*_ and *l*_2*j*_, and *n*_1_ and *n*_2_ are the number of samples in NS1 and NS2. This metric results in values ranging from zero (neighbor sets are identical) progressing theoretically to infinity as average differences increase.

Reptile endemism was measured using the CWE index (Crisp et al. 2001). The CWE of a location is calculated as the weighted endemism (WE; eqn 3) divided by species richness to correct for WE being high due to a high species richness (eqn 4; Gaston et al. 1998; Crisp et al. 2001; Linder 2001; Jetz et al. 2004).


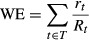
(3)


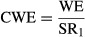
(4)

where *t* is a taxon in the set of taxa *T* in NS1, *r*_*t*_ is the local range of taxon *t* (the number of cells containing taxon *t* across NS1 and NS2) and *R*_*t*_ is the global range of taxon *t* across the entire data set, that is, the total number of cells in which it is found. SR_1_ is the species richness in NS1. Thus, CWE is the mean per-species range restriction to a cell or a set of cells in a neighborhood (Laffan et al. 2013).

Sample redundancy (eqn 5; Garcillan et al. 2003) measures the sampling effort of species observations across an area. It enables an assessment of reliability because redundancy and turnover patterns can be compared:


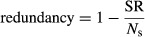
(5)

where SR is the species richness and *N*_s_ is the number of observations of each species in both neighbor sets. A value of zero indicates no redundancy in the sampling because there is only one observation per species. Higher redundancy values approaching a maximum value of one indicate areas that are well sampled because there are many observations for each species.

### GLM correlations as a function of spatial extent, location, and direction

The climatic turnover surfaces were standardized prior to analyzing their relationships with species turnover and CWE. Generalized linear model (GLM) analyses (Nelder and Wedderburn 1972) were separately applied to the results generated by each rotation of the moving window using either untransformed species turnover or CWE as the response variable and the corresponding four climatic turnover surfaces as predictor variables (i.e., one model per window orientation). This enabled the directional variation in reptile–environment relationships to be compared. A Gaussian error distribution for reptile turnover and CWE was assumed.

The GLM analyses were applied at a national extent and also with the study area stratified into different regional and local-scale extents. The regional-scale analyses used approximations of the five regional-scale biogeographic units delineated by the frameworks of Craw (1989) and Gibbs (2006) (Fig. 1B). Thus, two regions SN and MS in the southern NI and mid-SI (respectively) represented regions that are regarded as comparatively biologically depauperate. The remaining three regions (N, C, and S) in the northern NI and northern and southern SI, respectively, represented regions of higher diversity and endemism.

The local extent GLM analyses were applied using five of the smaller-scale area divisions devised by Crosby et al. (1998). This framework divides New Zealand into 29 discrete geographic regions that are widely used for the grouping, documentation and retrieval of specimens from a variety of taxonomic collections. The five Crosby et al. (1998) areas used were Northland, Wellington, Marlborough, mid-Canterbury, and Otago (Fig. 1C). Each of these locales is located within one of the five larger biogeographic regions of New Zealand. Thus, Northland, Marlborough, and Otago are, respectively, located within zones N, C, and S (high diversity/endemism). Wellington and mid-Canterbury are located within zones SN and MS (biologically depauperate).

Neither the regional nor local-scale biotic provinces described above were defined in order to describe the biogeographic patterns of New Zealand reptiles. However, their definition considered climatic and geographic features which may be pertinent to reptiles because they group regions with similar ecological and environmental characteristics. As yet, there is no definition of biotic provinces for New Zealand reptiles. Nonetheless, using these particular regional and local-scale provinces to partition New Zealand into different units seems appropriate given that these bioregions have climatic and geographic precedents for other species groups. For example, the division of the North Island at the Taupo line (separating zones N and SN at ∼39°S) is relevant because it is a recognized biogeographic barrier that delineates the distributions of several taxa, including reptiles.

Note that the use of overlapping moving windows introduces some spatial autocorrelation into the turnover surfaces. However, all the GLMs for each analysis extent will be equally affected by this bias; hence, the relative order of the biophysical relationships will not change.

## Results

### Correlogram

**Figure 2:**
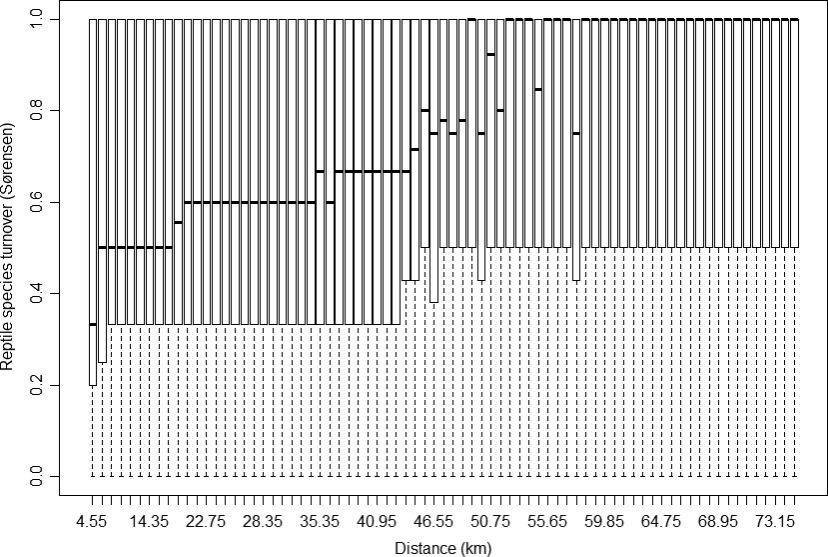
Correlogram summarizing the rate of change of species turnover of New Zealand reptiles as distances between pairs of cells increases. The correlogram is depicted as boxplots showing the distribution of turnover values between pairs of cells over geographic distance. Hence, for each boxplot, the black horizontal bar denotes the median turnover between pairs of cells separated by that distance, while the top and bottom of each box represent the 75th and 25th percentiles, respectively. The whiskers represent the minimum and maximum across all pairs at that separation distance. The distance on the *x*-axis at which the median turnover values begin to plateau is the maximum distance to which there is spatially structured turnover and was used to calibrate the dimensions of moving window analyses.

The key information provided by the correlogram in Figure 2 is shown by the median values at each geographic separation distance (shown by the thick, black horizontal bars) and how these values change with increasing separation distance. These values change abruptly due to the general sparseness of the reptile observations. The median reptile turnover values start to plateau at a distance of ∼45 km and at a Sørensen value of ∼0.8. This is the maximum distance to which there is spatially structured turnover. The larger neighbor set of the moving window (NS2) was therefore defined with major and minor radii of 45 km and 20 km, respectively. The length of the smaller neighbor set (NS1) was 20 km.

### Reptile species turnover, endemism, and sample redundancy

**Figure 3:**
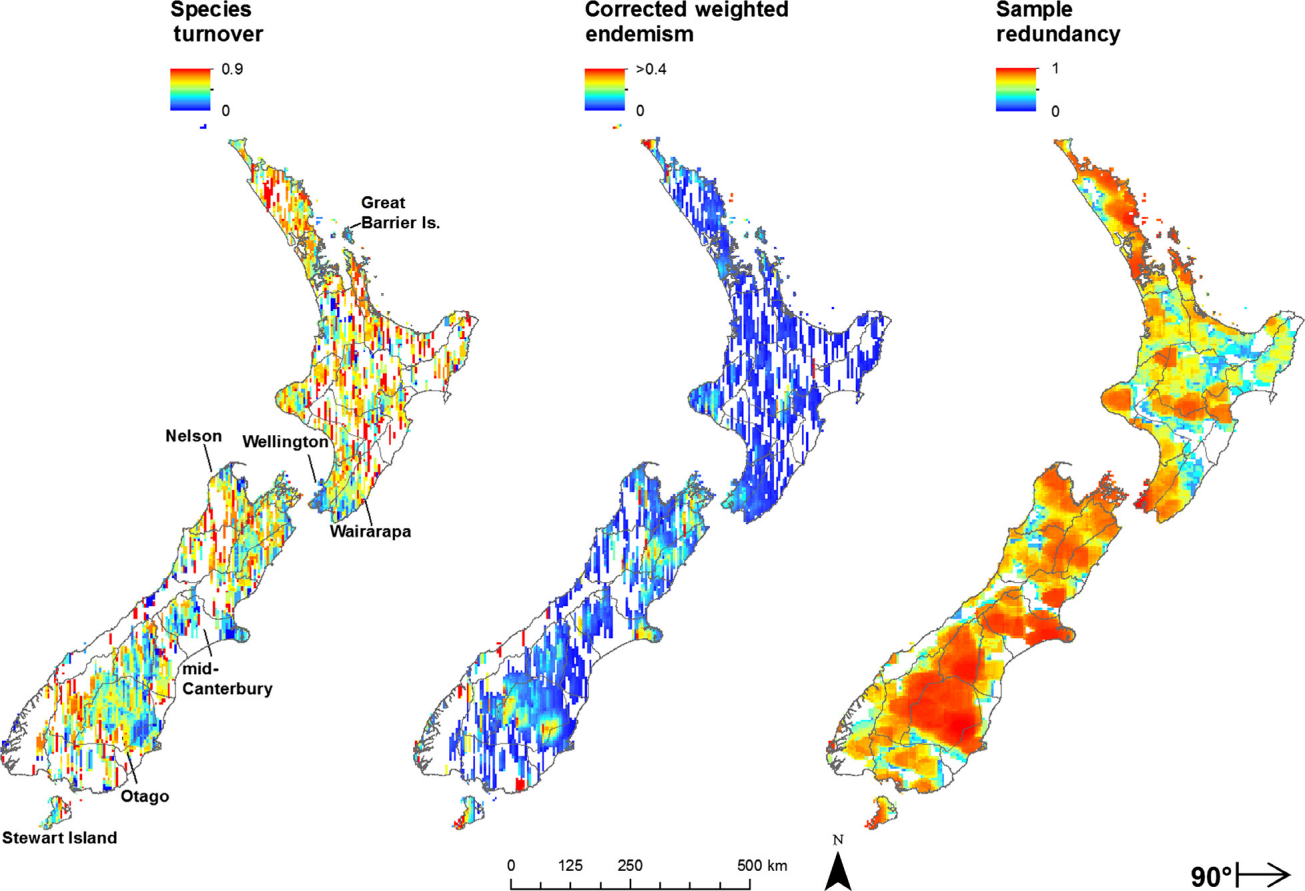
Reptile species turnover, corrected weighted endemism (CWE), and sample redundancy across New Zealand. The arrow (not drawn to scale) in the bottom right corner represents the overall orientation of the moving window. Note that the arrow base shows the orientation of neighbor set 1, with the arrow indicating the direction of neighbor set 2.

There is intricate variation in reptile species turnover across New Zealand (Fig. 3). Large areas of lower turnover are apparent in several locations, such as southern Wellington and Wairarapa in the southern NI, the SI north coast, and further south in Canterbury and Central Otago. Sampling gaps (white cells) are visible in several locations. Note that the parallel banding which is evident in the turnover map is an artifact of the moving window analysis, specifically the size of the window neighborhoods. Using smaller moving windows reduced the banding, but still revealed the same general pattern of turnover and endemism results (see Supporting Information Figure S1).

Low species turnover areas (Sørensen values < 0.3) comprise <11% (29,430 km^2^) of New Zealand and are distributed throughout the country. These areas are richly populated with diverse reptile communities, as they contain >50% of reptile observations (*n* = 5268) in 72 species. High turnover areas (values > 0.7), comprise 13% (33,740 km^2^) of New Zealand and comprise ∼6% of reptile observations in 61 species.

Corrected weighted endemism (CWE) values range from 0 to 1, but mean CWE is only 0.07 (SD = 0.09), with some variation depending upon moving window orientation (Fig. 3). There is also fine-scale variation in CWE patterns. For instance, there are three distinct CWE “hotspots” in Central Otago, separated by two strips of lower endemism. Areas of higher CWE (values ≥ 0.4) are limited to western Stewart Island and smaller patches along the western SI coastline, Nelson, Marlborough, and Northland.

**Figure 4:**
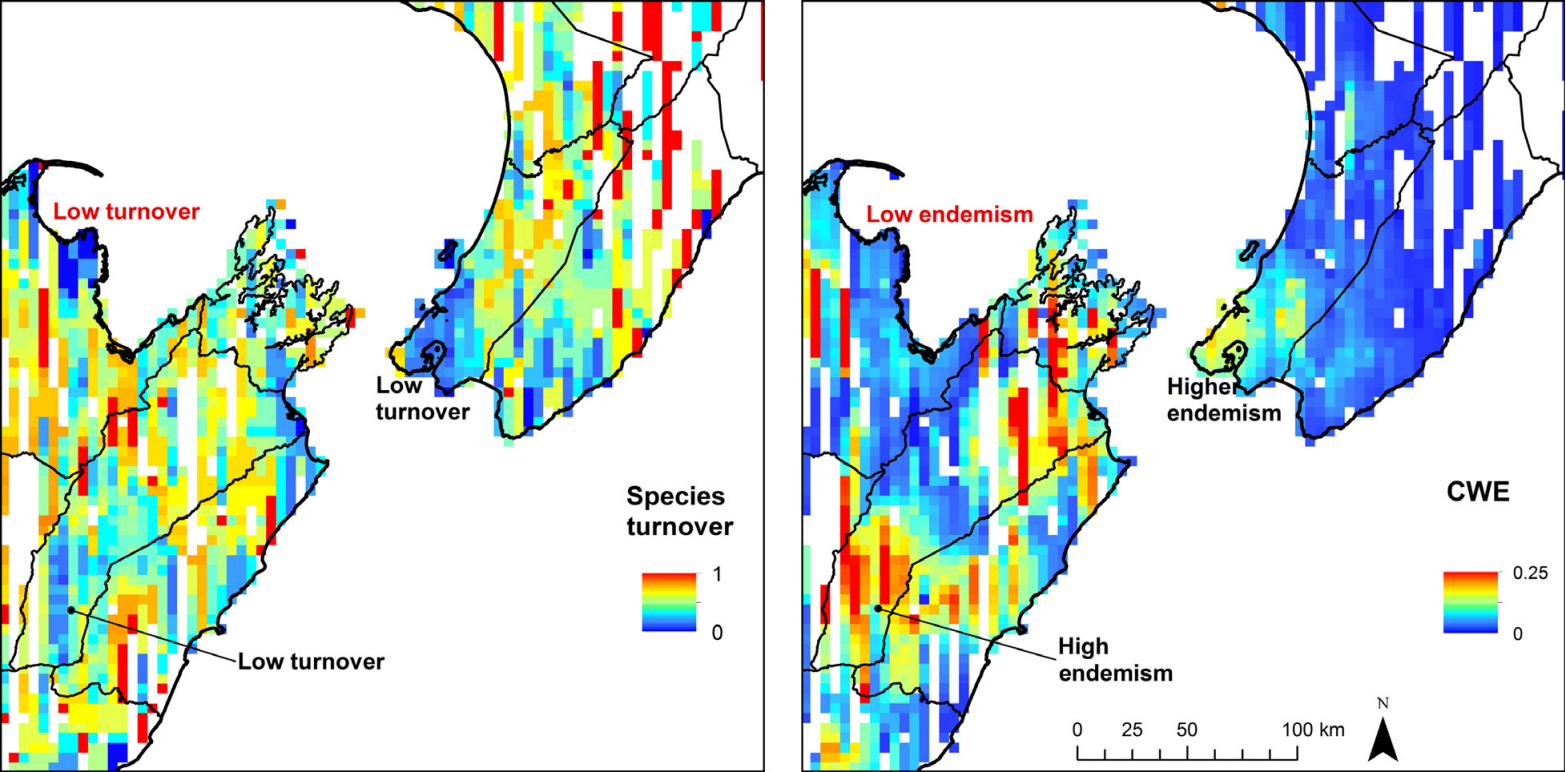
The relationship between reptile species turnover and corrected weighted endemism (CWE) varies depending upon geographic location (spatial nonstationarity), because low turnover is not always congruent with high CWE and vice versa.

There are some instances where distinct areas of high CWE co-occurred with low turnover zones, for example in southern Marlborough and southern Wellington (Fig. 4). In other regions, several low CWE areas correspond with low turnover zones, for example northern Nelson in the northern SI. This spatial nonstationarity in the relationships between CWE and species turnover patterns was reflected by their weak associations at the national analysis extent (*r*^2^ = 0.05, *P* < 0.01). Their associations were also weak at the regional scale. For example, relationships between the two biogeographic regions separated by Cook Straight [SN in the NI and C in the SI (Fig. 1B)] were weak and dissimilar: (*r*^2^ = 0.08, *P* < 0.01) and (*r*^2^ = 0.01, *P* < 0.01), respectively. In contrast, the local-scale analyses revealed much stronger species turnover–CWE associations in the corresponding areas either side of Cook Straight, that is, Wellington (*r*^2^ = 0.25, *P* < 0.01) and Marlborough (*r*^2^ = 0.24, *P* < 0.01).

The sample redundancy map shows adequate sampling redundancy across most of New Zealand (Fig. 3) and redundancy patterns do not correspond to the species turnover and CWE patterns. For instance, fine-scale variation in species turnover or endemism in Central Otago contrasts with comparatively uniform sample redundancy in this area. Areas of both high and low species turnover occur in locations where the sample redundancy is high.

### Reptile–climatic relationships

At the national extent, the models explained only a small proportion of the variation in species turnover and CWE, with the highest overall *r*^2^ being 0.05, which was identified by the moving window orientated at 330°. Nationally, turnover in either annual mean temperature or mean diurnal range consistently exerted the greatest influence on species turnover and CWE across all analysis orientations. For example, in the model with the highest overall *r*^2^ value, annual mean temperature turnover had the largest effect on CWE in terms of the magnitude of its standardized regression slope (*β*_1_ = 0.17, *P* < 0.001). Mean diurnal range and annual mean precipitation turnover were the only other predictors with a significant effect in this particular model, with respective standardized regression slopes of *β*_1_ = −0.06 and *β*_1_ = −0.04 (both *P* < 0.001). All models at the national extent were significant (*P* < 0.001). The high statistical significance will to some extent reflect the large number of observations.

Generally, the predictive capacity of models was also low at the regional analysis extents, with models producing a mean *r*^2^ value of 0.05 (SD = 0.04), although the mid-SI region (zone “MS” in Fig. 1B) was an exception. Within this region On average models explained 13% (SD = 0.04) of the variation in species turnover. Table 2 shows the magnitude of correlates' standardized regression slopes for the most predictive model within each region. Typically, a specific climatic predictor had a markedly larger effect on species turnover or CWE in each model in terms of the magnitude of its standardized regression slope. However, In contrast to the national-scale results, the identity of the climatic predictor with the greatest effect on species turnover or CWE varied with analysis direction and location. Thus, in two regions (the southern NI and adjacent northern SI) the influence of mean diurnal range and annual mean temperature turnover was greatest in 84% and 72% of directions, respectively (Table 3). By comparison, in the northern NI, mid-SI, and southern SI turnover in either annual precipitation or precipitation seasonality had the largest effect on species turnover in the majority of directions (96%, 92%, and 68%, respectively). This pattern of results was less consistent in the CWE-climatic models, and their correlates typically had shallower standardized regression slopes. Models were not significant in 3% of cases.

**Table 2:** The magnitude of the standardized regression slopes (*β*_1_) generated by the models that are most predictive of species turnover and endemism (i.e. for the model with the highest *r*^2^ coefficient of determination values) at each regional-scale location

	Taupo line, northern NI (N)				Taupo line, southern NI (SN)				Northern SI (C)				Mid-SI (MS)				Southern SI (S)			
					
	Turnover		CWE		Turnover		CWE		Turnover		CWE		Turnover		CWE		Turnover		CWE	
										
Correlate	*β*_1_	*r*^2^	*β*_1_	*r*^2^	*β*_1_	*r*^2^	*β*_1_	*r*^2^	*β*_1_	*r*^2^	*β*_1_	*r*^2^	*β*_1_	*r*^2^	*β*_1_	*r*^2^	*β*_1_	*r*^2^	*β*_1_	*r*^2^
Annual mean temp.	0.20	0.10	−0.10	0.13	−*0.01*	0.07	−0.14	0.10	**0.43**	0.06	**0.15**	0.05	**0.66**	0.22	**0.22**	0.08	−*0.01*	0.06	−0.16	0.06
Mean diurnal range	−0.34		−0.09		−**0.45**		0.10		*0.08*		*0.01*		0.27		−0.07		−0.12		*0.00*	
Annual precipitation	**0.48**		−0.08		*0.10*		**0.17**		−0.12		−0.08		−0.59		−*0.01*		**0.42**		**0.36**	
Precipitation seasonality	*0.02*		**0.14**		−0.15		−0.05		−*0.04*		−*0.03*		−0.57		0.21		−*0.06*		*0.03*	

CWE, corrected weighted endemism; N, northern North Island; NI, North Island; SN, southern North Island; C, northern South Island (“Central New Zealand”); MS, mid-South Island; SI, South Island; S, southern South Island (Fig. 1B).

Entries in italic font are not significant (*P* > 0.001). Entries in bold font denote the correlate with the largest regression slope.

**Table 3:** The number/proportion of directions in which the magnitude of the standardized regression slope of each climatic predictor was greatest at each regional-scale location

	Taupo line, northern NI		Taupo line, southern NI		Northern SI		Mid-SI		Southern SI	
					
Correlate	N	%	N	%	N	%	N	%	N	%
Annual mean temp.					18	72			8	32
Mean diurnal range	1	4	21	84	7	28				
Annual precipitation	24	96	4	16			2	8	17	68
Precipitation seasonality							23	92		

NI, North Island; SI, South Island.

At local extents, turnover in the climatic variables was considerably more predictive of species turnover and CWE. For instance, the most predictive model was for CWE in mid-Canterbury in the middle of the SI (*r*^2^ = 0.65, *P* < 0.001), which was identified by the moving window orientated at 165°. Table 4 shows the standardized regression slopes for the most predictive model at each locale. Again, the relative importance of temperature or precipitation turnover on reptile biodiversity varies with location. In terms of the complete range of models at each locale, annual mean temperature turnover consistently has the largest influence on species turnover in all directions in Northland in the northern NI. However, in the remaining four locales, across all analysis orientations three to four different climatic predictors have the greatest influence on species turnover (Table 5), instead of a maximum of two at regional scale. The CWE–climate relationships show a similar level of variability. As with the broader scale results, CWE–climatic turnover relationships differed to those of species-climatic turnover and typically the standardized regression slopes of the former were shallower.

**Table 4:** The magnitude of the standardized regression slopes (*β*_1_) generated by the models that are most predictive of species turnover and endemism (i.e. for the model with the highest *r*^2^ coefficient of determination values) at each local-scale location

	Northland (N)				Wellington (SN)				Marlborough (C)				Mid-Canterbury (MS)				Otago (S)			
					
	Turnover		CWE		Turnover		CWE		Turnover		CWE		Turnover		CWE		Turnover		CWE	
										
Correlate	*β*_1_	*r*^2^	*β*_1_	*r*^2^	*β*_1_	*r*^2^	*β*_1_	*r*^2^	*β*_1_	*r*^2^	*β*_1_	*r*^2^	*β*_1_	*r*^2^	*β*_1_	*r*^2^	*β*_1_	*r*^2^	*β*_1_	*r*^2^
Annual mean temp.	0.62	0.22	*0.13*	0.26	−*0.31*	0.38	−0.15	0.62	0.25	0.24	−0.08	0.18	**1.14**	0.34	−0.17	0.65	**0.64**	0.18	−0.25	0.33
Mean diurnal range	−*0.04*		−*0.08*		−**0.70**		**0.16**		0.20		0.10		−*0.11*		**0.37**		−0.30		0.25	
Annual precipitation	−0.31		−**0.45**		0.59		*0.05*		−**0.29**		**0.14**		−0.83		0.17		−0.47		**0.41**	
Precipitation seasonality	**0.71**		0.41		0.37		−0.07		*0.08*		*0.00*		−0.44		0.21		−0.24		*0.03*	

CWE, corrected weighted endemism; N, northern North Island; SN, southern North Island; C, northern South Island (“Central New Zealand”); MS, mid-South Island; S, southern South Island (Fig.1C).

Entries in italic font are not significant (*P* > 0.001). Entries in bold font denote the correlate with the largest regression slope.

**Table 5:** The number/proportion of directions in which the magnitude of the standardized regression slope of each climatic predictor was greatest at each local-scale location.

	Northland		Wellington		Marlborough		Mid-Canterbury		Otago	
					
Correlate	N	%	N	%	N	%	N	%	N	%
Annual mean temp.	25	100	3	12	9	36	10	40	10	40
Mean diurnal range			9	36	7	28	8	32	3	12
Annual precipitation			2	8	5	20	3	12	12	48
Precipitation seasonality			11	44	4	16	4	16		

Other patterns were also more apparent at local scales. The relative importance of each climatic predictor on species turnover and CWE showed a directional trend. The effect of temperature-related or precipitation-related turnover often tended to be strongest in 4–5 sequential directions. As the moving window continued to rotate through 360°, the relative importance of temperature or precipitation predictors frequently switched in a systematic manner, such that a different predictor type had the greatest effect in the next batch of directions. This is shown for two example locations (Wellington and Otago) in Table S1 in the Supporting Information. Additionally, in localities regarded as high in species diversity/endemism (Northland, Marlborough, and Otago), model predictive capacity was weaker relative to biologically depauperate localities (Wellington and mid-Canterbury). Local scale models were not significant (*P* > 0.001) in 2.5% of cases.

## Discussion

We now consider the geographic variation in reptile species turnover, CWE and climatic turnover relationships and the implications that these results may have for reptiles and potentially other species groups elsewhere in the world.

### Spatial dependencies of reptile–climatic relationships

Climatic turnover, which is analogous to climatic heterogeneity, had weak predictive capacity at the national scale. This was also generally the case at the regional analysis scale. In contrast, climatic turnover was substantially more predictive of species turnover and CWE at local scales. The relative influence of each climatic predictor on reptile biodiversity showed greater variation between the different local-scale areas in New Zealand and there was also marked anisotropy in the reptile–climatic relationships. This spatial variation (nonstationarity) in reptile–climatic relationships was possibly more apparent at local scales because the influence of climatic turnover varied within a single locale due to fine-scale geographic variability (e.g., variations in landforms may mediate climatic influences). The rotation of the moving window detected the mediatory effects of this fine-scale geographic variability at local scales. In contrast, the influence of these fine-scale geographic variations was more concealed at the national and regional analysis scales, despite the high analysis resolution used. At these broader spatial extents, reptile–climatic relationships possibly appeared less variable because at these scales the combined effects of a multitude of different fine-scale geographic influences effectively offset one another. Future investigations may extend these descriptive explanations with a quantitative analysis of how variation in different landforms might mediate climatic effects and their influence on reptile biodiversity.

Previous studies that have focused on the broad-scale or geographically invariant, isotropic dependencies between reptile diversity and environmental conditions have found that temperature is consistently more predictive of reptile ecological patterns than precipitation (Rodriguez et al. 2005; Aragon et al. 2010; Qian 2010; Buckley et al. 2012). We found this to be the case only at the national scale, where turnover in either of the temperature predictors consistently had the greatest effect on species turnover and CWE. However, the magnitude of the influence of temperature and precipitation-related turnover varied with respect to location and direction in local-scale areas (and to a lesser degree in the regional-scale areas). Why might variability in rainfall patterns exert a marked influence on reptile compositional turnover? Reptiles must attain near-optimal body temperature in order to maximize physiological performance, which they may seek to maintain in response to thermal variability via thermoregulation. Higher rainfall may result in fewer opportunities for behavioral thermoregulation relative to drier conditions, perhaps by constraining reptiles to suboptimal habitats (Clusella-Trullas et al. 2011). Heat transfer from the physical environment can also be reduced by high precipitation and cloud cover, thus restricting reptile activity periods and lowering body temperature (Marquis et al. 2008). High precipitation is also associated with elevated extinction risk in New Zealand reptiles (Tingley et al. 2013). Thus, while reptile species turnover and CWE patterns are dependent upon temperature-related turnover, variation in rainfall also appears to have an important influence. Presumably, rainfall heterogeneity may affect reptile phenologies, which in turn may eventually affect population dynamics and so the rate of species turnover. A complex interaction between precipitation and temperature turnover may occur, which is an important question not addressed by our study. Nonetheless, our results caution against assuming that variability in temperature-related variables will always be most predictive of reptile biodiversity across all spatial scales and locations.

### Data quality

The degree to which these findings might be influenced by sampling bias/data quality issues requires consideration. As with any macroecological data set covering a broad geographic area, the reptile data used here have limitations such as an incomplete collection effort and by being presence-only data as opposed presence-absence. Nonetheless, these limitations do not mean that the data set is inappropriate for the spatial analyses that we have performed, nor that it cannot be used for any form of inference. A degree of collection bias will be a feature of this data (and indeed any data set). However, 54% (*n* = 5639 of 10,477 observations) of the lizard sample points are >1 km from any road (either surfaced or unsurfaced). We repeated the species turnover and endemism analyses using only this subset of the data and the same moving window analyses. Overall, the species turnover and endemism surfaces appear similar (see Supporting Information Figure S2). Note that 42% of records (*n* = 4377) are >2 km from all roads. Consequently, we conclude that this is the best available data set for this research and that its quality does not invalidate the analyses used.

### Spatial nonstationarity of reptile species turnover and CWE

Low species turnover areas did not consistently co-occur in the same locations as areas of high CWE, and overall correlations between species turnover and CWE patterns were weak across all analysis extents, with the exception of Wellington and Marlborough at local scales. The relative influence of each climatic predictor on species turnover and CWE differed in an inconsistent manner. CWE-climatic correlate pairs also typically had shallower standardized regression slopes than species turnover-climatic correlates. More generally, as low species turnover areas in New Zealand host large, diverse reptile communities, they may be regarded as conservation priorities. However, such a prioritization might not equate to conservation of highly endemic reptile species.

### Biophysical relationships in biologically rich and depauperate areas

Our secondary aim was to determine whether reptile–climatic relationships systematically differed among different zone types, that is, between regions regarded as biologically rich or depauperate. In the biologically rich northern NI (zone N), annual precipitation turnover has the largest effect on species turnover in 96% of directions compared with 4% for mean diurnal range. In the neighboring “biologically poor” southern NI (zone SN), the influence of mean diurnal range turnover is largest in 84% of directions, compared with 16% for annual precipitation. Thus, the species–climatic relationships seem to “switch” in the two adjacent biogeographic regions separated by the Taupo Line at ∼39°S. The Taupo line is a recognized biogeographic barrier. It delineates the distributions of several biota such as most skink species in the *Oligosoma* genus (∼53 species), because only two species occur continuously across the NI (McCann 1956; Towns et al. 1985; Pickard and Towns 1988).

Other differences in reptile biodiversity patterns and their relationships with climatic turnover were only apparent at local scales. Associations between Reptile turnover, CWE and climatic turnover were generally weaker in areas within biogeographic regions regarded as high in biodiversity. In contrast, these relationships were typically stronger within the biologically depauperate regions.

### Links to historical climatic and geographic processes

Qualitatively, the variation in reptile species turnover and CWE patterns may reflect recognized phylogeographic disjunctions. A biogeographic break is evident in eastern and western Otago in the species turnover and CWE maps. Comparably, intraspecific genetic breaks in eastern and western Otago have been found between populations of *Oligosoma maccanni* (O'Neill et al. 2008), *Oligosoma otagense* (Chapple et al. 2012), *Oligosoma grande* (Berry and Gleeson 2005), and *Oligosoma chloronoton* (Greaves et al. 2007).

Higher CWE and low species turnover were apparent either side of Cook Straight, between southern Wellington in the NI and Marlborough in the northern SI. Both locations also showed the strongest CWE–species turnover associations, which were of similar magnitude. These similarities could reflect the historical presence of a land-bridge connecting the two islands ∼6–2 mya. Pleistocene glacial cycles created periodic land-bridges across Cook Straight (Lewis et al. 1994), which some species such as *Oligosoma zelandicum* (O'Neill et al. 2008) and *Oligosoma polychroma* (Liggins et al. 2008) may have traversed. Other reptile species occur on only one side of Cook Strait, such as *Oligosoma aeneum* and *Oligosoma ornatum* (Chapple et al. 2008).

Low turnover areas comprise <11% of New Zealand, yet they are populated with diverse reptile communities comprising >50% of reptile observations. These low turnover areas occur throughout the country, both in high biodiversity areas, such as Marlborough and Nelson in the northern SI, and also in areas characterized as biologically depauperate, for example mid-Canterbury (Wardle 1963, 1988; McGlone et al. 2001; Wallis and Trewick 2001). Marlborough and Nelson are regarded as possible glacial refugia for several taxa (Wardle 1963, 1988; McGlone et al. 2001) and may have afforded habitat suitable to reptiles during Pleistocene glaciation ∼2 mya (Bull and Whitaker 1975).

## Conclusions

Assessing how species might respond to environmental variability could provide insights into the targeting and planning of their conservation. We found that turnover in climatic predictors was substantially more predictive of reptile species turnover and endemism at local scales, whereas associations were generally weak at national and regional scales. Moreover, the variation in the magnitude of the effect of different climatic predictors was also most apparent at local scales. While temperature-related turnover consistently had the greatest effect on reptile biodiversity nationally, precipitation-related turnover had the greatest influence in several locations at regional and local scales. As the relative importance of climatic predictors on reptile species turnover and endemism exhibits marked spatial variation, the biophysical relationships discovered by broad-scale, spatially invariant analyses may not generalize across scales and locations.

The spatial dependences in reptile–climatic relationships were possibly more apparent at the local scales because the influence of climatic turnover varied substantially within a single region due to fine-scale geographic variability. These intricate, local-scale variations were either not apparent or more concealed at the national and regional analysis scales. The influence of climatic turnover on the compositional turnover and endemism of other species groups in other regions may exhibit similar degrees of geographic variation. Such variation might be discerned more readily if studies at broad scales are complemented by geographically variant, local-scale analyses.

## Data Accessibility

Reptile point locality data were derived from the New Zealand Department of Conservation Herpetofauna Database (BioWeb Herpetofauna Database 2012). This data is obtainable at: http://www.doc.govt.nz/conservation/native-animals/reptiles-and-frogs/herpetological-digital-resources/. Climatic data sets were downloaded from WorldClim (http://www.worldclim.org/) on 12 May 2013.

## Supporting Information

**Figure S1.** The prominence of parallel banding in the species turnover patterns is an artifact of the size of the moving window neighborhoods used in the analyses. Using a smaller window renders the banding less apparent, but the same turnover and endemism hotspots are still apparent.

**Figure S2.** Species turnover and endemism patterns generated with the entire data set (*n* = 10,477) and for a subset of the data set using only reptile observation points at least 1 km in distance from any road (surfaced or unsurfaced; *n* = 5639)

**Table S1.** The relative importance of each climatic predictor on species turnover and CWE showed a directional trend as the moving window analyses were rotated through 360°.
