# Supplementary material for: Spatial variation in the climatic predictors of species compositional turnover and endemism
Source: Ecol Evol. 2014 Jul 29;4(16):3264–78. doi: 10.1002/ece3.1156 (PMC4222213; doi:10.1002/ece3.1156)

**Supporting Information**

**Spatial variation in the climatic predictors of species compositional turnover and endemism**

Giovanni Di Virgilio, Shawn W. Laffan, Malte C. Ebach and David G. Chapple

**Figure S1** The prominence of parallel banding in the species turnover patterns is an artefact of the size of the moving window neighbourhoods used in the analyses. Using a smaller window (right panel) renders the banding less apparent, but the same turnover and endemism hotspots are still apparent.


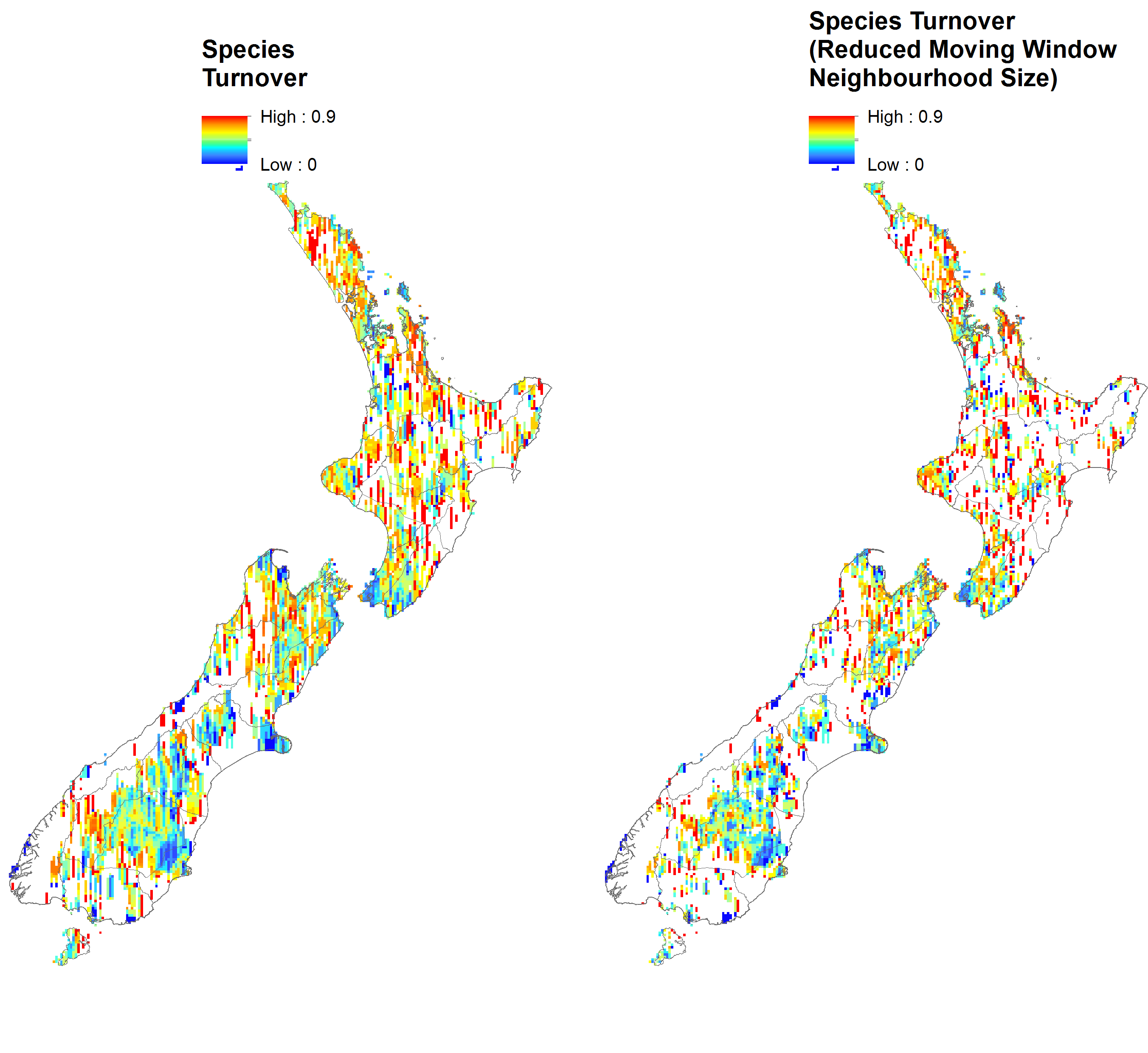

Supplement: Supplementary file 1 — Figure S1. The prominence of parallel banding in the species turnover patterns is an artifact of the size of the moving window neighborhoods used in the analyses. Using a smaller window renders the banding less apparent, but the same turnover and endemism hotspots are still apparent. [file ece30004-3264-sd1.doc]
