# Supplementary material for: Spatial variation in the climatic predictors of species compositional turnover and endemism
Source: Ecol Evol. 2014 Jul 29;4(16):3264–78. doi: 10.1002/ece3.1156 (PMC4222213; doi:10.1002/ece3.1156)

**Supporting Information**

**Spatial variation in the climatic predictors of species compositional turnover and endemism**

Giovanni Di Virgilio, Shawn W. Laffan, Malte C. Ebach and David G. Chapple

**Figure S2** Species turnover and endemism patterns generated with the entire data set (n = 10,477) and for a subset of the data set using only reptile observation points at least 1 km in distance from any road (surfaced or unsurfaced) (n = 5639)


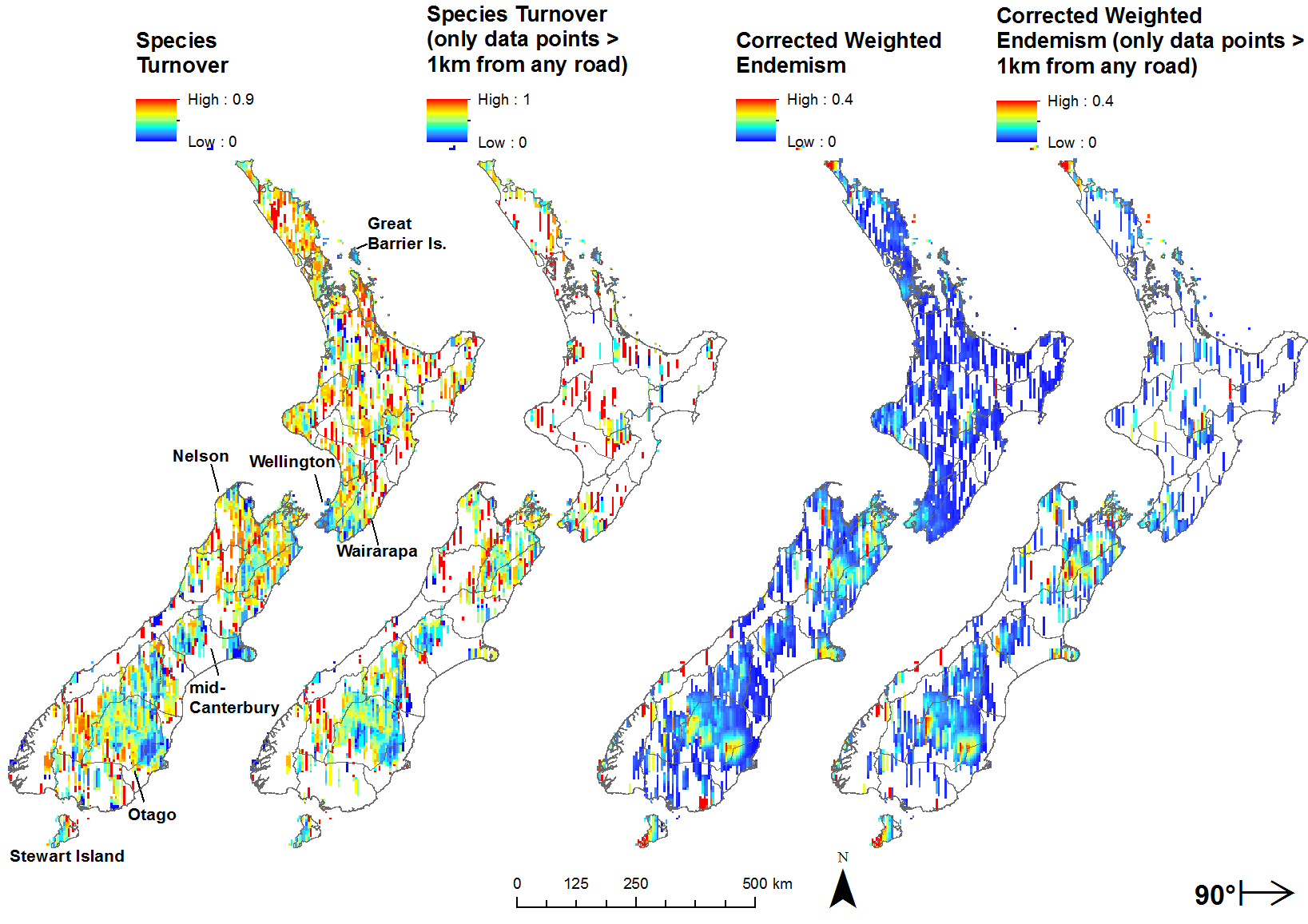

Supplement: Supplementary file 2 — Figure S2. Species turnover and endemism patterns generated with the entire data set (n = 10,477) and for a subset of the data set using only reptile observation points at least 1 km in distance from any road (surfaced or unsurfaced; n = 5639) [file ece30004-3264-sd2.doc]
