# Supplementary material for: Spatial variation in the climatic predictors of species compositional turnover and endemism
Source: Ecol Evol. 2014 Jul 29;4(16):3264–78. doi: 10.1002/ece3.1156 (PMC4222213; doi:10.1002/ece3.1156)
Supplement: Supplementary file 3 — Table S1. The relative importance of each climatic predictor on species turnover and CWE showed a directional trend as the moving window analyses were rotated through 360°. [file ece30004-3264-sd3.doc]

**Supporting Information**

**Spatial variation in the climatic predictors of species compositional turnover and endemism**

Giovanni Di Virgilio, Shawn W. Laffan, Malte C. Ebach and David G. Chapple

**Table S1**

| The relative importance of each climatic predictor on species turnover and CWE showed a directional trend as the moving window analyses were rotated through 360°. Two example locations at the local analysis extent are shown below. | | | | | | | | | | |
| --- | --- | --- | --- | --- | --- | --- | --- | --- | --- | --- |
|  |  |  |  |  |  |  |  |  |  |  |
| Wellington (Species Turnover) | | | | |  | Otago (Endemism) | | | | |
|  |  |  |  |  |  |  |  |  |  |  |
| Correlate | β1 | P | r^2^ | Window Angle (°) |  | Correlate | β1 | P | r^2^ | Window Angle (°) |
| BIO01 | 0.39 | 0.01 | 0.36 | 0 |  | BIO01 | -0.26 | 0.00 | 0.26 | 0 |
| **BIO02** | -0.66 | 0.00 |  |  |  | BIO02 | 0.18 | 0.00 |  |  |
| BIO12 | 0.23 | 0.12 |  |  |  | **BIO12** | 0.33 | 0.00 |  |  |
| BIO15 | 0.18 | 0.06 |  |  |  | BIO15 | 0.03 | 0.02 |  |  |
| BIO01 | -0.01 | 0.95 | 0.33 | 15 |  | BIO01 | -0.28 | 0.00 | 0.24 | 15 |
| **BIO02** | -0.65 | 0.00 |  |  |  | BIO02 | 0.21 | 0.00 |  |  |
| BIO12 | 0.40 | 0.00 |  |  |  | **BIO12** | 0.33 | 0.00 |  |  |
| BIO15 | 0.17 | 0.18 |  |  |  | BIO15 | 0.03 | 0.05 |  |  |
| BIO01 | -0.31 | 0.02 | 0.38 | 30 |  | BIO01 | -0.28 | 0.00 | 0.23 | 30 |
| **BIO02** | -0.70 | 0.00 |  |  |  | BIO02 | 0.25 | 0.00 |  |  |
| BIO12 | 0.59 | 0.00 |  |  |  | **BIO12** | 0.29 | 0.00 |  |  |
| BIO15 | 0.37 | 0.01 |  |  |  | BIO15 | 0.01 | 0.52 |  |  |
| BIO01 | -0.43 | 0.01 | 0.29 | 45 |  | BIO01 | -0.26 | 0.00 | 0.20 | 45 |
| **BIO02** | -0.72 | 0.00 |  |  |  | BIO02 | 0.23 | 0.00 |  |  |
| BIO12 | 0.67 | 0.00 |  |  |  | **BIO12** | 0.29 | 0.00 |  |  |
| BIO15 | 0.15 | 0.35 |  |  |  | BIO15 | 0.02 | 0.31 |  |  |
| BIO01 | -0.20 | 0.25 | 0.27 | 60 |  | BIO01 | -0.19 | 0.00 | 0.23 | 60 |
| **BIO02** | -1.00 | 0.00 |  |  |  | **BIO02** | 0.30 | 0.00 |  |  |
| BIO12 | 0.36 | 0.06 |  |  |  | BIO12 | 0.23 | 0.00 |  |  |
| BIO15 | 0.04 | 0.80 |  |  |  | BIO15 | 0.03 | 0.09 |  |  |
| BIO01 | -0.45 | 0.04 | 0.19 | 75 |  | BIO01 | -0.11 | 0.00 | 0.22 | 75 |
| **BIO02** | -0.55 | 0.00 |  |  |  | **BIO02** | 0.26 | 0.00 |  |  |
| BIO12 | 0.51 | 0.03 |  |  |  | BIO12 | 0.20 | 0.00 |  |  |
| BIO15 | 0.43 | 0.01 |  |  |  | BIO15 | 0.03 | 0.06 |  |  |
| BIO01 | 0.21 | 0.38 | 0.13 | 90 |  | BIO01 | 0.01 | 0.79 | 0.21 | 90 |
| BIO02 | -0.41 | 0.04 |  |  |  | **BIO02** | 0.24 | 0.00 |  |  |
| BIO12 | -0.23 | 0.37 |  |  |  | BIO12 | 0.13 | 0.00 |  |  |
| **BIO15** | 0.60 | 0.00 |  |  |  | BIO15 | 0.02 | 0.10 |  |  |
| BIO01 | -0.19 | 0.39 | 0.09 | 105 |  | BIO01 | 0.03 | 0.32 | 0.12 | 105 |
| BIO02 | -0.21 | 0.26 |  |  |  | **BIO02** | 0.15 | 0.00 |  |  |
| BIO12 | 0.04 | 0.88 |  |  |  | BIO12 | 0.08 | 0.00 |  |  |
| **BIO15** | 0.68 | 0.00 |  |  |  | BIO15 | 0.01 | 0.33 |  |  |
| BIO01 | 0.11 | 0.55 | 0.11 | 120 |  | BIO01 | 0.06 | 0.07 | 0.06 | 120 |
| BIO02 | -0.41 | 0.06 |  |  |  | **BIO02** | 0.11 | 0.00 |  |  |
| BIO12 | -0.08 | 0.69 |  |  |  | BIO12 | 0.02 | 0.50 |  |  |
| **BIO15** | 0.60 | 0.01 |  |  |  | BIO15 | -0.02 | 0.11 |  |  |
| BIO01 | -0.09 | 0.62 | 0.18 | 135 |  | BIO01 | 0.01 | 0.83 | 0.03 | 135 |
| BIO02 | -0.55 | 0.00 |  |  |  | **BIO02** | 0.06 | 0.00 |  |  |
| BIO12 | 0.05 | 0.82 |  |  |  | BIO12 | 0.03 | 0.19 |  |  |
| **BIO15** | 0.92 | 0.00 |  |  |  | BIO15 | -0.03 | 0.03 |  |  |
| BIO01 | 0.28 | 0.08 | 0.20 | 150 |  | BIO01 | 0.01 | 0.84 | 0.04 | 150 |
| BIO02 | -0.18 | 0.21 |  |  |  | **BIO02** | 0.06 | 0.00 |  |  |
| BIO12 | -0.14 | 0.44 |  |  |  | BIO12 | 0.04 | 0.11 |  |  |
| **BIO15** | 0.74 | 0.00 |  |  |  | BIO15 | -0.03 | 0.06 |  |  |
| BIO01 | 0.17 | 0.29 | 0.09 | 165 |  | BIO01 | -0.06 | 0.09 | 0.05 | 165 |
| BIO02 | 0.15 | 0.24 |  |  |  | **BIO02** | 0.09 | 0.00 |  |  |
| **BIO12** | 0.19 | 0.37 |  |  |  | BIO12 | 0.10 | 0.00 |  |  |
| BIO15 | 0.05 | 0.78 |  |  |  | BIO15 | -0.02 | 0.14 |  |  |
| BIO01 | 0.09 | 0.57 | 0.11 | 180 |  | BIO01 | -0.10 | 0.00 | 0.07 | 180 |
| **BIO02** | 0.27 | 0.01 |  |  |  | BIO02 | 0.12 | 0.00 |  |  |
| BIO12 | 0.25 | 0.19 |  |  |  | **BIO12** | 0.14 | 0.00 |  |  |
| BIO15 | 0.06 | 0.72 |  |  |  | BIO15 | -0.02 | 0.17 |  |  |
| BIO01 | -0.09 | 0.66 | 0.16 | 195 |  | BIO01 | -0.19 | 0.00 | 0.12 | 195 |
| BIO02 | 0.34 | 0.01 |  |  |  | BIO02 | 0.18 | 0.00 |  |  |
| **BIO12** | 0.42 | 0.07 |  |  |  | **BIO12** | 0.20 | 0.00 |  |  |
| BIO15 | 0.26 | 0.14 |  |  |  | BIO15 | -0.04 | 0.02 |  |  |
| BIO01 | 0.30 | 0.09 | 0.21 | 210 |  | **BIO01** | -0.26 | 0.00 | 0.20 | 210 |
| BIO02 | 0.33 | 0.02 |  |  |  | BIO02 | 0.24 | 0.00 |  |  |
| BIO12 | -0.01 | 0.95 |  |  |  | BIO12 | 0.24 | 0.00 |  |  |
| **BIO15** | 0.59 | 0.01 |  |  |  | BIO15 | -0.04 | 0.01 |  |  |
| BIO01 | 0.31 | 0.15 | 0.25 | 225 |  | **BIO01** | -0.29 | 0.00 | 0.23 | 225 |
| BIO02 | 0.41 | 0.01 |  |  |  | BIO02 | 0.23 | 0.00 |  |  |
| BIO12 | 0.05 | 0.86 |  |  |  | BIO12 | 0.29 | 0.00 |  |  |
| **BIO15** | 0.67 | 0.00 |  |  |  | BIO15 | -0.01 | 0.57 |  |  |
| **BIO01** | 0.59 | 0.00 | 0.26 | 240 |  | **BIO01** | -0.34 | 0.00 | 0.24 | 240 |
| BIO02 | 0.21 | 0.21 |  |  |  | BIO02 | 0.24 | 0.00 |  |  |
| BIO12 | -0.09 | 0.70 |  |  |  | BIO12 | 0.33 | 0.00 |  |  |
| BIO15 | 0.56 | 0.00 |  |  |  | BIO15 | 0.01 | 0.31 |  |  |
| BIO01 | 0.34 | 0.03 | 0.31 | 255 |  | BIO01 | -0.33 | 0.00 | 0.24 | 255 |
| BIO02 | -0.02 | 0.92 |  |  |  | BIO02 | 0.24 | 0.00 |  |  |
| BIO12 | 0.30 | 0.11 |  |  |  | **BIO12** | 0.38 | 0.00 |  |  |
| **BIO15** | 0.48 | 0.00 |  |  |  | BIO15 | 0.02 | 0.28 |  |  |
| BIO01 | 0.52 | 0.01 | 0.32 | 270 |  | BIO01 | -0.25 | 0.00 | 0.33 | 270 |
| BIO02 | -0.06 | 0.78 |  |  |  | BIO02 | 0.25 | 0.00 |  |  |
| BIO12 | 0.12 | 0.56 |  |  |  | **BIO12** | 0.41 | 0.00 |  |  |
| **BIO15** | 0.57 | 0.00 |  |  |  | BIO15 | 0.03 | 0.04 |  |  |
| BIO01 | 0.19 | 0.42 | 0.22 | 285 |  | BIO01 | -0.16 | 0.00 | 0.27 | 285 |
| BIO02 | -0.24 | 0.34 |  |  |  | BIO02 | 0.18 | 0.00 |  |  |
| BIO12 | 0.32 | 0.19 |  |  |  | **BIO12** | 0.32 | 0.00 |  |  |
| **BIO15** | 0.42 | 0.00 |  |  |  | BIO15 | 0.04 | 0.00 |  |  |
| BIO01 | 0.40 | 0.04 | 0.26 | 300 |  | BIO01 | -0.06 | 0.10 | 0.26 | 300 |
| BIO02 | -0.05 | 0.86 |  |  |  | BIO02 | 0.17 | 0.00 |  |  |
| BIO12 | 0.12 | 0.48 |  |  |  | **BIO12** | 0.25 | 0.00 |  |  |
| **BIO15** | 0.42 | 0.00 |  |  |  | BIO15 | 0.05 | 0.00 |  |  |
| **BIO01** | 0.49 | 0.01 | 0.23 | 315 |  | BIO01 | -0.06 | 0.06 | 0.20 | 315 |
| BIO02 | -0.21 | 0.41 |  |  |  | BIO02 | 0.14 | 0.00 |  |  |
| BIO12 | -0.02 | 0.92 |  |  |  | **BIO12** | 0.21 | 0.00 |  |  |
| BIO15 | 0.45 | 0.00 |  |  |  | BIO15 | 0.05 | 0.00 |  |  |
| **BIO01** | 0.48 | 0.00 | 0.30 | 330 |  | BIO01 | -0.11 | 0.00 | 0.20 | 330 |
| BIO02 | -0.46 | 0.03 |  |  |  | BIO02 | 0.14 | 0.00 |  |  |
| BIO12 | 0.16 | 0.29 |  |  |  | **BIO12** | 0.24 | 0.00 |  |  |
| BIO15 | 0.29 | 0.00 |  |  |  | BIO15 | 0.04 | 0.01 |  |  |
| BIO01 | 0.50 | 0.01 | 0.30 | 345 |  | BIO01 | -0.19 | 0.00 | 0.26 | 345 |
| **BIO02** | -0.78 | 0.00 |  |  |  | BIO02 | 0.17 | 0.00 |  |  |
| BIO12 | 0.26 | 0.15 |  |  |  | **BIO12** | 0.31 | 0.00 |  |  |
| BIO15 | 0.10 | 0.34 |  |  |  | BIO15 | 0.04 | 0.00 |  |  |
| BIO01 | 0.39 | 0.01 | 0.36 | 360 |  | BIO01 | -0.26 | 0.00 | 0.26 | 360 |
| **BIO02** | -0.66 | 0.00 |  |  |  | BIO02 | 0.18 | 0.00 |  |  |
| BIO12 | 0.23 | 0.12 |  |  |  | **BIO12** | 0.33 | 0.00 |  |  |
| BIO15 | 0.18 | 0.06 |  |  |  | BIO15 | 0.03 | 0.02 |  |  |
